# Acute kidney injury in *Staphylococcus aureus* bacteraemia: a multicentre cohort study of incidence, timing, recovery and associations with antibiotic treatment

**DOI:** 10.1093/jac/dkag304

**Published:** 2026-09-02

**Authors:** F S Sinkeler, I J E Kouijzer, N G L Jager, J ten Oever, E Leegwater, J Gisolf, D Reuling, Z M Spoel, M P M Hensgens, R J Brüggemann

**Affiliations:** Department of Pharmacy, Pharmacology and Toxicology, Radboud University Medical Center, Nijmegen, The Netherlands; Department of Internal Medicine and Radboudumc Community for Infectious Diseases, Radboud University Medical Center, Nijmegen, The Netherlands; Department of Pharmacy, Pharmacology and Toxicology, Radboud University Medical Center, Nijmegen, The Netherlands; Department of Internal Medicine and Radboudumc Community for Infectious Diseases, Radboud University Medical Center, Nijmegen, The Netherlands; Department of Pharmacy, Pharmacology and Toxicology, Radboud University Medical Center, Nijmegen, The Netherlands; Department of Internal Medicine, Rijnstate Hospital, Arnhem, The Netherlands; Department of Internal Medicine, University Medical Center Utrecht, Utrecht, The Netherlands; Department of Internal Medicine, University Medical Center Utrecht, Utrecht, The Netherlands; Department of Internal Medicine, University Medical Center Utrecht, Utrecht, The Netherlands; Julius Center for Health Sciences and Primary Care, University Medical Center Utrecht, Utrecht, The Netherlands; Department of Pharmacy, Pharmacology and Toxicology, Radboud University Medical Center, Nijmegen, The Netherlands

## Abstract

**Background and objectives:**

*Staphylococcus aureus* bacteraemia (SAB) is associated with high mortality and frequent complications, including acute kidney injury (AKI). Although flucloxacillin has been associated with higher AKI rates than cefazolin, the clinical characteristics of AKI, including severity, timing, recovery and the potential influence of dosing, remain unclear.

**Methods:**

We conducted a retrospective multicentre cohort study across three hospitals in the Netherlands, including adults with SAB treated with cefazolin or flucloxacillin. AKI incidence and severity were compared using adjusted regression analyses. Time-to-event and cumulative incidence analyses were used to evaluate AKI timing and renal recovery. Among flucloxacillin-treated patients, the association between initial dose and AKI was evaluated.

**Results:**

Among 1408 patients, 483 (34.3%) developed AKI. Most AKI episodes occurred early after index blood culture (median 1.3 days), and recovery within 30 days decreased with increasing AKI severity (81.7% in Stage 1 versus 50.5% in Stage 3). AKI occurred more frequently among patients treated with flucloxacillin than cefazolin (466/1324 [35.2%] versus 17/84 [20.2%], *P* = 0.004). After multivariable adjustment, flucloxacillin remained associated with higher AKI risk (adjusted OR 2.37, 95% CI 1.30–4.55). Among patients with AKI, severity distribution did not differ between treatments (*P* = 0.599). Initial flucloxacillin dose was not associated with AKI occurrence, timing or severity.

**Conclusions:**

Flucloxacillin was associated with increased AKI risk compared with cefazolin, with most AKI episodes occurring early after index blood culture and recovery decreasing with increasing AKI severity. Lower flucloxacillin doses were not associated with reduced AKI risk, suggesting that dose reduction alone may not mitigate flucloxacillin-associated nephrotoxicity.

## Background

*Staphylococcus aureus* bacteraemia (SAB) is associated with high mortality and a broad range of complications, including acute kidney injury (AKI).^1–3^ AKI in SAB arises through multiple mechanisms—including sepsis-related inflammation, haemodynamic instability and drug-induced nephrotoxicity—and is strongly linked to adverse outcomes such as chronic kidney disease and death.^3,4^

The timing of AKI onset may provide insight into potential underlying mechanisms. Early AKI frequently occurs in the context of sepsis-related inflammation and haemodynamic disturbances, and typically occurs within the first week of infection,^5^ whereas drug-induced forms of AKI generally develop later, often 7–10 days after drug exposure.^5,6^ However, substantial overlap exists between these mechanisms, particularly in patients with sepsis receiving potentially nephrotoxic therapies. Characterizing the timing of AKI development and the subsequent course of renal function is therefore important for clinical management.

Cefazolin and flucloxacillin, the latter being an anti-staphylococcal penicillin (ASP), are first-line agents for treating SAB. Several observational studies have reported higher AKI rates with ASPs compared with cefazolin, with adjusted analyses indicating an increased risk of AKI associated with (flu)cloxacillin use.^2,7,8^ More recently, the multicentre randomized S. aureus Network Adaptive Platform (SNAP) trial reported AKI in 19.6% of flucloxacillin-treated patients compared with 13.9% of those receiving cefazolin.^9^ Similarly, the CloCeBa trial demonstrated AKI rates of 12% in patients treated with cloxacillin compared with 1% in those receiving cefazolin.^10^ Taken together, these findings have established an increased risk of AKI associated with flucloxacillin compared with cefazolin. However, important questions regarding AKI severity, timing of onset, renal recovery and the potential contribution of flucloxacillin dosing to AKI risk in SAB remain unanswered.

To address these knowledge gaps, we aimed to characterize AKI during SAB, including its incidence, timing, severity and renal recovery in patients treated with cefazolin or flucloxacillin. We further sought to explore whether the initial flucloxacillin dose was associated with AKI occurrence.

## Methods

### Study population

This retrospective observational cohort study was conducted at two university hospitals [Radboud University Medical Center (Radboudumc), Nijmegen and University Medical Center Utrecht (UMC Utrecht), Utrecht] and one non-university teaching hospital (Rijnstate, Arnhem) in the Netherlands. The Medical Ethics Committees of all participating centres waived the need for ethical approval (Radboudumc: 2024-17864; UMC Utrecht: 24U-1444 and Rijnstate: 205-2621).

All adults (≥18 years) with at least one positive *S. aureus* blood culture and at least one serum creatinine measurement during admission were eligible for inclusion, comprising patients admitted between January 2013 and July 2024 in Radboudumc, January 2014 and December 2023 in UMC Utrecht and January 2009 and December 2018 in Rijnstate. For patients with multiple SAB episodes, only the first episode was analysed. Exclusion criteria comprised blood culture contamination, pre-existing chronic renal replacement therapy (RRT), methicillin-resistant *S. aureus* (MRSA) and withdrawal of consent for use of clinical data. Contamination was defined as *S. aureus* growth considered not reflective of a true infectious episode. The final study population was restricted to patients receiving cefazolin or flucloxacillin as first-line therapy; patients with AKI before the index blood culture were subsequently excluded.

Patients who received at least one dose of cefazolin or flucloxacillin were assigned to treatment groups according to the first administered antibiotic. Treatment groups were not reclassified according to subsequent antibiotic switches or treatment duration. Cefazolin administrations given solely as (extended) perioperative prophylaxis or as ‘pro re nata’ (as needed) administrations were not considered treatment initiation.

### Data collection

All clinical data were obtained from electronic patient files (UMC Utrecht) or by using data mining software CTcue (Rijnstate and Radboudumc; CTcue B.V., Amsterdam, the Netherlands). For CTcue-extracted data, 10 randomly selected records were manually reviewed to validate extraction accuracy.

Microbiological data were used to identify positive blood cultures for *S. aureus*. Extracted variables included demographics, medical history, microbiological results, antibiotic treatment, concomitant medication during admission and laboratory results. Concomitant nephrotoxic medication was based on known nephrotoxic agents associated with AKI and was defined as receipt of at least one dose of vancomycin, any aminoglycoside, any 5-aminosalicylates, allopurinol, any calcineurin inhibitor, any immune checkpoint inhibitor, co-trimoxazole, any diuretic, lithium, methotrexate, any non-steroidal anti-inflammatory drugs, any proton pump inhibitor, any renin-angiotensin system-inhibitor, any salicylates >100 mg, any sodium-glucose cotransporter-2-inhibitor (SGLT2 inhibitors) and (val)acyclovir.^11^ For the UMC Utrecht cohort, exposure to SGLT2 inhibitors and immune checkpoint inhibitors was not available in the administration data and therefore not included in the analysis.

### Definitions

The index blood culture was defined as the first blood culture yielding *S. aureus*, irrespective of the clinical location or timing within the hospital stay. Individual baseline serum creatinine was defined using a hierarchical approach. First, the median outpatient creatinine 100–90 days before admission was used; if unavailable, the median from 365-7 days before admission was used. If no pre-admission creatinine measurements were available, baseline creatinine was estimated based on age and sex according to Pottel *et al.*^12^

AKI was staged according to the Kidney Disease: Improving Global Outcomes (KDIGO) criteria, applied to all serum creatinine measurements obtained after the index blood culture during admission. Stage 1 AKI was defined as an increase in serum creatinine of 1.5–1.9 times baseline or an absolute increase in serum creatinine ≥26.5 µmol/L (≥0.3 mg/dL). Stage 2 was defined as an increase in serum creatinine of 2.0–2.9 times baseline. Stage 3 was classified as an increase in serum creatinine of 3.0 times baseline, an increase in serum creatinine to ≥353.6 µmol/L (≥4.0 mg/dL) or the initiation of renal replacement therapy.^13^ Urine output criteria were not considered, as urinary output was not routinely measured. The timing of AKI onset was defined as the first creatinine measurement fulfilling KDIGO criteria relative to the date of the index blood culture. Early AKI was defined as onset ≤7 days and late AKI as onset >7 days after obtaining the index blood culture, based on all creatinine measurements obtained during the hospital admission. Recovery of kidney function was defined as a return of creatinine to <1.5 times the baseline value and to ≤26.5 µmol/L above baseline within 30 days of AKI onset.

Comorbidities were classified according to Charlson comorbidity index (CCI).^14^ Healthcare-associated infection was defined as any non-community-acquired infection, including both hospital-acquired and healthcare-associated infections. Thirty-day mortality, length of hospital admission and intensive care unit (ICU) admission were calculated from the date of the index blood culture.

Infection complications were classified into six predefined categories: line-related, skin and soft tissue infection (SSTI), osteoarticular, endocarditis, central nervous system (CNS) and other foci, based on anatomical location or device involvement. A binary variable indicated the presence of one or more relevant foci. Patients could be classified into multiple categories. In the Rijnstate cohort, SSTI and CNS foci were not systematically collected and were therefore not included in the respective variables for that centre.

Flucloxacillin dosing was evaluated only in patients who received flucloxacillin as initial therapy. The initial daily dose was defined as the first documented 24-h dosing regimen after treatment initiation. Dose regimens were classified as 6, 8, 12 g/day or ‘other’. For descriptive analyses, the 6-g and 8-g categories were combined into a low-dose group, whereas the 12-g category constituted the high-dose group. Patients in the ‘other’ category were excluded from dose–AKI analyses.

### Outcome measures

The primary outcome was AKI during hospitalization, including severity (KDIGO Stages 1–3) and timing of onset, assessed in relation to first-line antibiotic treatment with cefazolin or flucloxacillin. Secondary outcomes included the association between initial flucloxacillin dose and AKI occurrence, severity and timing of onset. The tertiary outcome was renal recovery among patients who developed AKI, evaluated according to severity stage.

### Statistical analyses

Continuous variables were summarized as medians with interquartile ranges (IQRs) and categorical variables as counts and percentages. AKI incidence and severity (KDIGO Stages 1–3) were compared between cefazolin- and flucloxacillin-treated patients using Fisher’s exact test. Differences in AKI timing (no AKI, early AKI ≤7 days, late AKI >7 days) were assessed using the chi-squared test. A multivariable logistic regression model was used to evaluate the association between first antibiotic and AKI occurrence, adjusting for age, sex, baseline creatinine, CCI, ICU admission, acquisition type, hospital, number of nephrotoxic co-medications and vancomycin and aminoglycoside use, which were included separately because of their well-established nephrotoxic potential. AKI timing was further analysed using multinomial logistic regression with the same covariates, estimating odds ratios (ORs) for early and late AKI relative to no AKI. Results are reported as ORs with 95% confidence intervals (CIs).

AKI incidence rates per 100 patient-days from the index blood culture until hospital discharge were calculated overall and stratified by hospital and initial antibiotic treatment. Among patients receiving flucloxacillin as initial therapy, a multivariable logistic regression model was used to assess the association between initial flucloxacillin dose (6–8 g/day versus 12 g/day) and AKI occurrence, adjusting for the same covariates as the primary analysis, with additional adjustment for endocarditis.

Time to AKI (KDIGO stage ≥1) within 30 days of the index blood culture was analysed using cumulative incidence functions, treating death before AKI as a competing event. Follow-up was censored at hospital discharge or Day 30, whichever occurred first. Kaplan–Meier analysis was performed for the composite outcome of AKI (KDIGO stage ≥1) or death within 30 days of the index blood culture, stratified by initial flucloxacillin dose. Differences between Kaplan–Meier curves were assessed using the log-rank test. Renal recovery among patients with AKI was evaluated using cumulative incidence functions, with recovery as the event of interest and death before recovery treated as a competing event. Follow-up for recovery analyses was limited to 30 days after AKI onset or the last creatinine measurement, whichever came first. A two-sided *P* value <0.05 was considered statistically significant.

## Results

### Demographics

**Figure 1.**
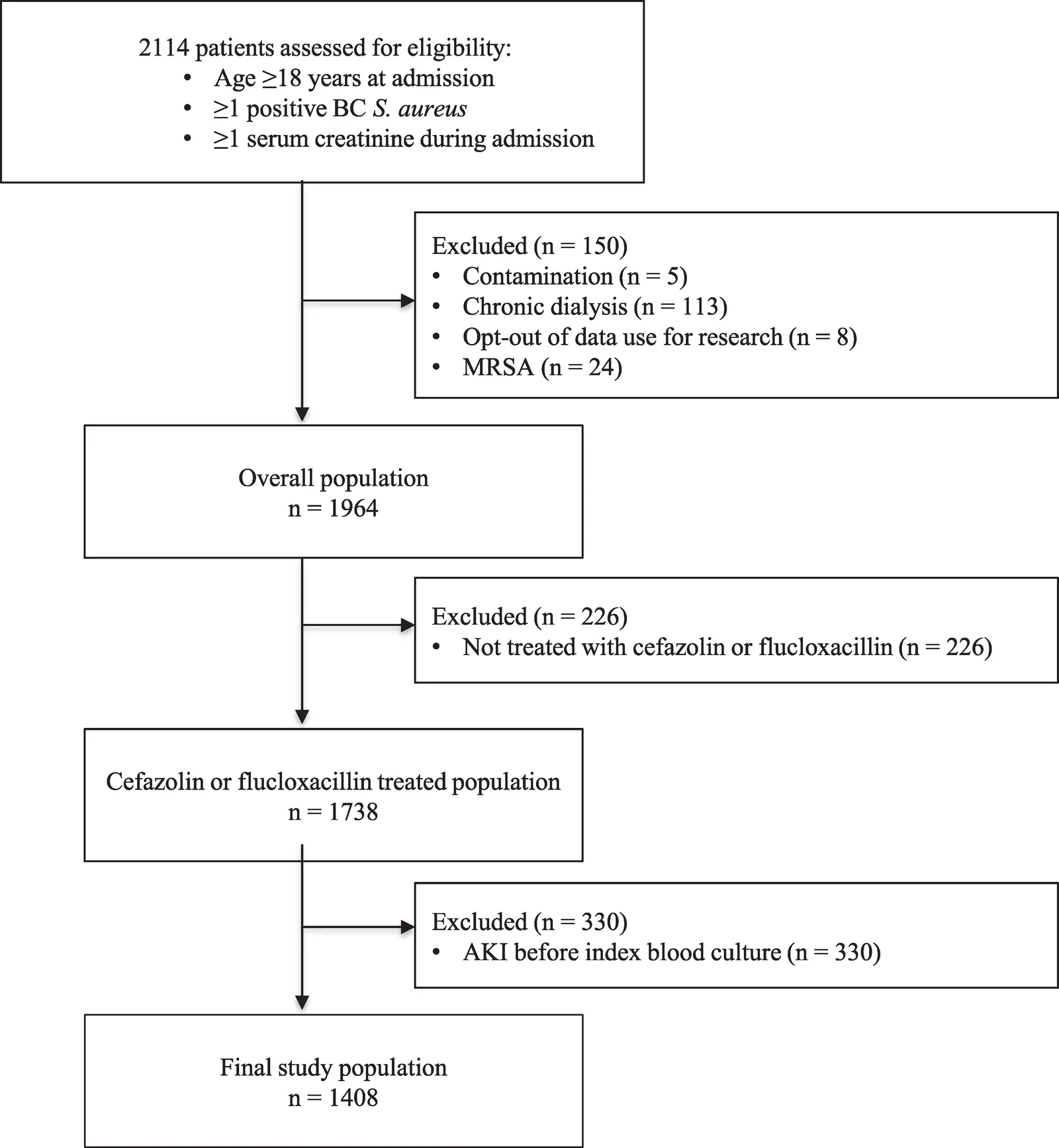
Study flow diagram. Flow diagram of patient inclusion and exclusion. Adult patients with at least one positive blood culture for *Staphylococcus aureus* and at least one serum creatinine measurement during admission were eligible. After application of the study selection criteria, the final study population comprised 1408 patients treated with cefazolin or flucloxacillin.

In total, 2114 patients with SAB were identified. After applying the exclusion criteria and restricting the cohort to patients treated with cefazolin or flucloxacillin, 1408 patients were included in the final study population (Figure 1). Of these, 500 patients were admitted at Radboudumc, 503 at Rijnstate and 405 at UMC Utrecht.

ICU admission varied across centres, from 13.3% in UMC Utrecht to 22.0% in Radboudumc, with an intermediate rate in Rijnstate (16.3%). Comorbidity burden differed modestly, with median CCI scores of 3 in Radboudumc, 2 in Rijnstate and 3.5 in UMC Utrecht. Distribution of infection foci differed between centres, with osteoarticular infections being more common in Rijnstate (60.6%) compared with Radboudumc (22.2%) and UMC Utrecht (18.5%), whereas endocarditis frequency ranged from 5.1% in UMC Utrecht to 9.8% in Radboudumc and 16.3% in Rijnstate. Of the 1408 included patients, 84 (6.0%) received cefazolin and 1324 (94.0%) received flucloxacillin as first-line therapy. During admission, 212 patients (15.1%) received both cefazolin and flucloxacillin; 187 (88.2%) started with flucloxacillin. The median duration of antibiotic treatment was 13 days (IQR 7–17), with 11 days (IQR 5–15) for patients starting on cefazolin and 13 days (IQR 7–17) for those starting on flucloxacillin. Cohort descriptions are shown in Table 1.

**Table 1.** Cohort description

	Overall population *n* = 1408	Flucloxacillin treated *n* = 1324	Cefazolin treated *n* = 84
Male	882 (62.6%)	836 (63.1%)	46 (54.8%)
Age (years)	67 (55–76)	67 (55–76)	66 (57–73)
BMI (kg/m^2^)	25.5 (22.8–28.8)	25.5 (22.8–28.8)	26.0 (23.0–28.8)
**Comorbidities**			
CCI score^a^	3 (1–5)	3 (1–5)	4 (2–5.8)
**Laboratory parameters at baseline** ^b^			
CRP (mg/L)	113 (30–231)	114 (29–231)	107 (50–230)
Leukocytes (×10^9^/L)	11.2 (7.9–15.6)	11.2 (8.0–15.6)	11.1 (7.7–16.2)
**Acquisition**			
Community-associated	623 (44.4%)	592 (44.8%)	31 (36.9%)
Healthcare-associated	781 (55.6%)	728 (55.2%)	53 (63.1%)
**Infection complications** ^c^			
Line-related	317 (23.3%)	296 (23.2%)	21 (25.9%)
Skin and soft tissue	215 (25.1%)	200 (25.2%)	15 (23.8%)
Osteoarticular	482 (35.5%)	461 (36.1%)	21 (25.9%)
Endocarditis	151 (10.8%)	140 (10.7%)	11 (13.1%)
Central nervous system	17 (2.0%)	16 (2.0%)	1 (1.6%)
Other	673 (49.5%)	628 (49.1%)	45 (55.6%)
**Nephrotoxic co-medication**			
Use of vancomycin	99 (7.0%)	91 (6.9%)	8 (9.5%)
Use of aminoglycosides	281 (20.0%)	268 (20.2%)	13 (15.5%)
Number of concomitant agents			
0	225 (16.0%)	218 (16.5%)	7 (8.3%)
1	331 (23.5%)	316 (23.9%)	15 (17.9%)
2	426 (30.3%)	397 (30.0%)	29 (34.5%)
≥3	426 (30.3%)	393 (29.7%)	33 (39.3%)
**Outcome**			
Admission (days)	20 (13–29)	19 (13–29)	25 (15–38)
ICU admission^d^	246 (18.7%)	233 (18.9%)	13 (16.5%)
30-Day mortality	201 (14.3%)	197 (14.8%)	5 (6.0%)

Data are presented as number (%) or median (IQR).

AKI, acute kidney injury; BMI, body mass index; CCI, Charlson comorbidity index; CRP, C-reactive protein; ICU, intensive care unit.

^a^CCI data were missing for 149 patients from UMC Utrecht.

^b^Baseline CRP and leukocyte counts were only available for Radboudumc and Rijnstate.

^c^SSTI and CNS foci were not recorded in Rijnstate. At Rijnstate, 9 patients had only line-related infection recorded. At UMC Utrecht, infection foci were missing for 30 patients, and only endocarditis was recorded for 75 patients.

^d^ICU admission data were missing for 95 patients from UMC Utrecht.

### Acute kidney injury

Baseline creatinine was based on creatinine values from 100 to 90 days prior to admission in 156 patients, from values obtained 365 to 7 days before admission in 796 patients and was estimated based on age and sex in 456 patients. The distribution of measured and estimated baseline creatinine categories did not differ between treatment groups (*P* = 0.911).

As shown in Table 2, baseline and peak creatinine levels were comparable between cefazolin- and flucloxacillin-treated patients. Among AKI patients, 48 (9.9% of all AKI cases) reached their peak creatinine level on the day of index blood culture collection. AKI occurred more frequently in the flucloxacillin group compared with cefazolin (35.2% versus 20.2%; *P* = 0.004), corresponding to a lower unadjusted odds of AKI with cefazolin (OR 0.47; 95% CI 0.25–0.82). After multivariable adjustment, flucloxacillin use remained independently associated with a higher risk of AKI compared with cefazolin (adjusted OR 2.37, 95% CI 1.30–4.55). This association remained similar in a sensitivity analysis restricted to patients with a measured baseline creatinine (adjusted OR 2.59, 95% CI 1.29–5.63). The distribution of AKI stages among patients who developed AKI did not differ significantly between treatment groups (*P* = 0.599).

**Table 2.** Renal function and acute kidney injury outcomes by initial antibiotic therapy

	Overall population *n* = 1408	Flucloxacillin treated *n* = 1324	Cefazolin treated *n* = 84
Number of creatinine measurements	7 (4–13)	7 (4–13)	8 (5–14)
Creatinine follow-up duration (days)^a^	13.3 (7.3–22.0)	13.4 (7.3–21.3)	13.3 (8.2–27.4)
Baseline creatinine (µmol/L)	79.6 (66.3–91.1)	79.6 (66.3–91.5)	79.6 (66.2–88.4)
Max creatinine during admission (µmol/L)	95.0 (73.0–144.0)	95.0 (73.0–148.0)	90.0 (67.8–124.5)
Presence of AKI	483 (34.3%)	466 (35.2%)	17 (20.2%)
**Severity of AKI (KDIGO stage)**			
1	268 (55.5%)	257 (55.2%)	11 (64.7%)
2	110 (22.8%)	108 (23.2%)	2 (11.8%)
3	105 (21.7%)	101 (21.7%)	4 (23.5%)
Start RRT during admission	23 (1.6%)	21 (1.6%)	2 (2.4%)
**Timing of AKI onset** ^b^			
≤ 7 days	388 (80.3%)	375 (80.5%)	13 (76.5%)
> 7 days	95 (19.7%)	91 (19.5%)	4 (23.5%)

Data are presented as number (%) or median (IQR).

AKI, acute kidney injury; KDIGO, Kidney Disease: Improving Global Outcomes; RRT, renal replacement therapy.

^a^Creatinine measurements were assessed from the index blood culture until hospital discharge; follow-up duration was calculated from the index blood culture to the last creatinine measurement during admission.

^b^Percentages for AKI timing were calculated among patients with AKI.

As a supplementary analysis accounting for differences in hospital stay duration, the AKI incidence rate was 0.84 per 100 patient-days in cefazolin-treated patients and 1.66 in flucloxacillin-treated patients. Among flucloxacillin-treated patients, incidence rates were similar across centres, ranging from 1.43 (UMC Utrecht) to 1.89 (Rijnstate) per 100 patient-days.

### Timing of AKI onset

**Figure 2.**
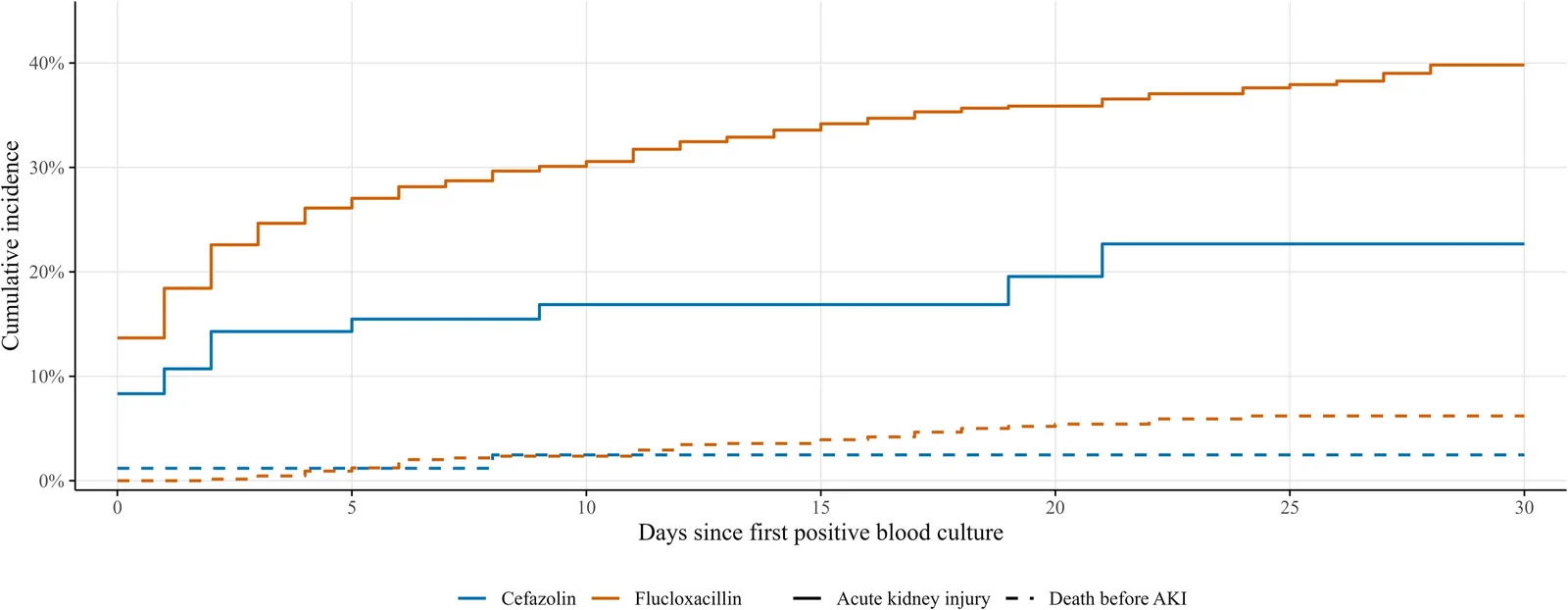
Cumulative incidence of acute kidney injury according to initial antibiotic treatment. Cumulative incidence functions for acute kidney injury and death before acute kidney injury within 30 days after the index blood culture, stratified by initial antibiotic treatment (cefazolin or flucloxacillin). Death before acute kidney injury was treated as a competing event.

Among patients who developed AKI, the median time from index blood culture to AKI onset was 1.3 days in both treatment groups, with similar distributions for cefazolin- and flucloxacillin-treated patients (IQR 0.4–4.7 and 0.4–5.0 days, respectively). The cumulative incidence of AKI over time according to initial antibiotic treatment is shown in Figure 2. The distribution of AKI timing (no AKI, early AKI ≤7 days, late AKI >7 days) differed significantly between treatment groups (*P* = 0.019). Early AKI occurred in 15.5% of cefazolin-treated patients and 28.3% of flucloxacillin-treated patients, whereas late AKI occurred in 4.8% and 6.9%, respectively.

In multinomial regression, flucloxacillin use showed higher odds for early AKI compared with cefazolin (OR 2.51, 95% CI 1.28–4.93; *P* = 0.007). There was no significant association between flucloxacillin use and late-onset AKI (OR 2.08, 95% CI 0.63–6.86; *P* = 0.23).

### Flucloxacillin dose

Among the 1324 patients who received flucloxacillin as first-line therapy, 718 (55.7%) started on a high-dose regimen (12 g/day), whereas 572 (44.3%) received a lower dose (6–8 g/day). An additional 34 patients (2.6%) had other dosing regimens. Distribution varied by centre, with high-dose initiation observed in 86.1% (396/460) of Radboudumc patients, 27.6% (134/485) of Rijnstate patients and 49.6% (188/379) of UMC Utrecht patients.

**Figure 3.**
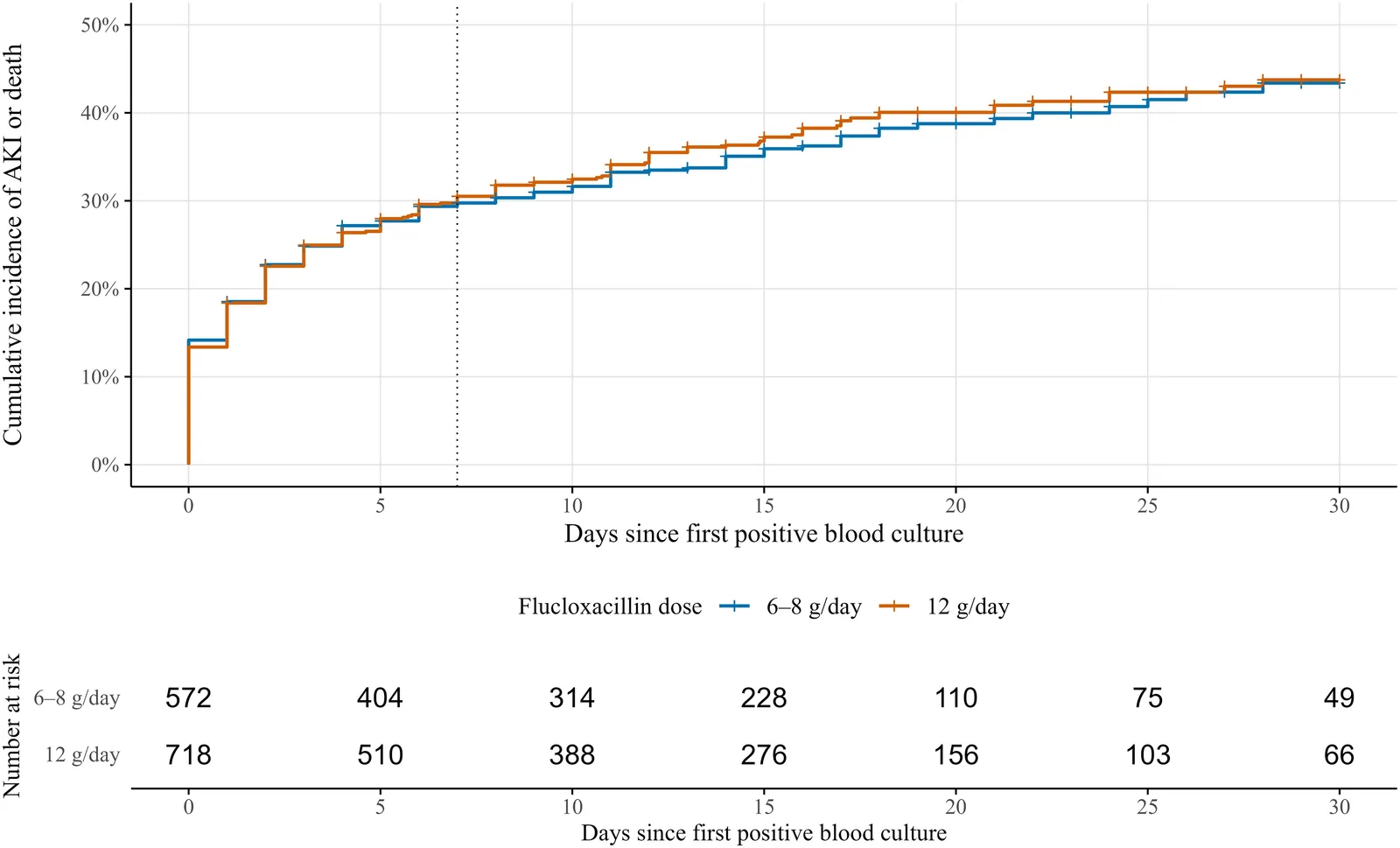
Time to AKI or death according to initial flucloxacillin dose. Kaplan–Meier curves for the composite outcome of acute kidney injury (KDIGO stage ≥1) or death within 30 days after the index blood culture, stratified by initial flucloxacillin dose (6–8 g/day versus 12 g/day). Tick marks indicate censoring, and numbers at risk are shown below the *x*-axis. The vertical dotted line indicates Day 7 after the index blood culture.

AKI incidence was similar between dosing groups: 35.2% (253/718) in patients receiving high-dose flucloxacillin versus 35.7% (204/572) in those receiving lower doses. Among patients who developed AKI, the distribution of KDIGO stages was similar between dosing groups (Stage 1: 52.2% versus 59.8%; Stage 2: 25.7% versus 20.1%; Stage 3: 22.1% versus 20.1%, respectively). The timing of AKI onset did not differ meaningfully by dosing strategy: median time from the index blood culture to AKI onset was 1.3 days (IQR 0–5.0) in the high-dose group and 1.5 days (IQR 0.6–4.7) in the low-dose group. Early AKI (≤7 days) occurred in 160/572 patients (28.0%) receiving 6–8 g/day and 206/718 patients (28.7%) receiving 12 g/day. After multivariable adjustment, high-dose flucloxacillin (12 g/day) was not associated with an increased risk of AKI compared with lower-dose regimens (6–8 g/day) (adjusted OR 0.85, 95% CI 0.62–1.17). Consistent with these findings, the Kaplan–Meier curves for the composite outcome of AKI or death within 30 days were nearly identical between dosing groups (log-rank *P* = 0.71; Figure 3).

### Recovery of AKI

**Figure 4.**
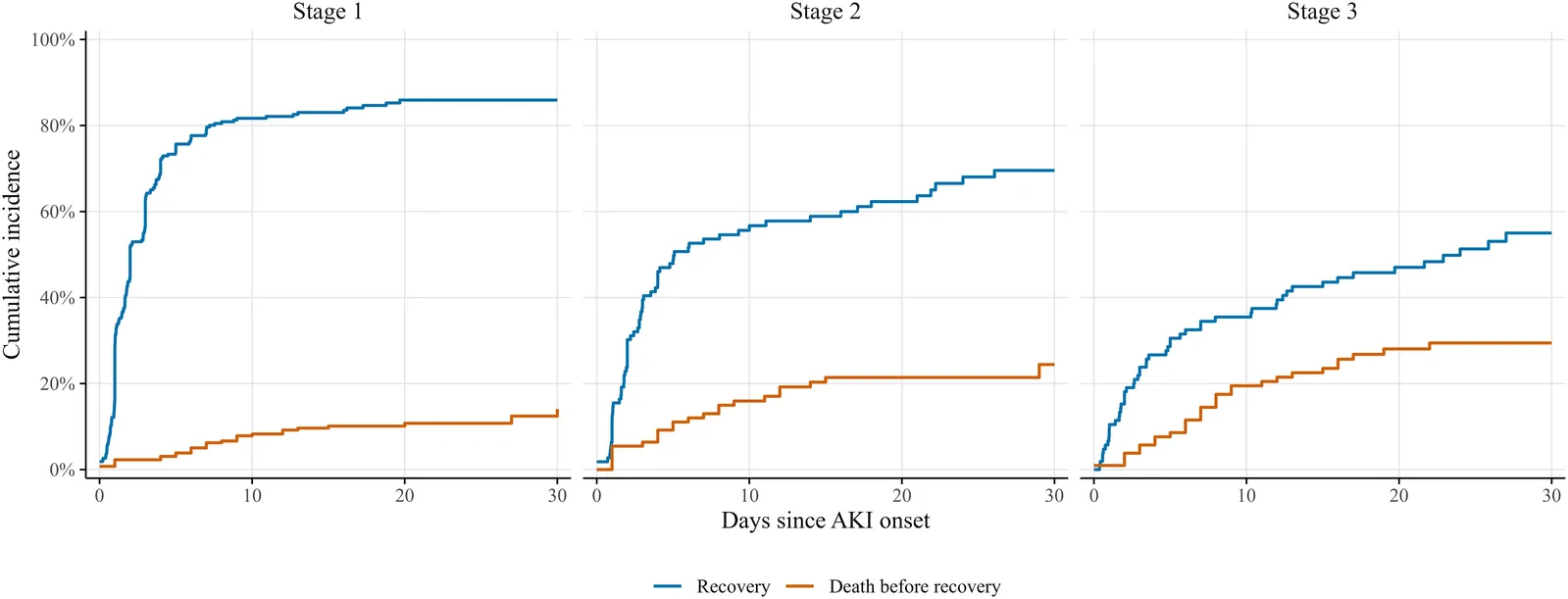
Renal recovery and death before recovery according to AKI severity. Cumulative incidence functions for renal recovery and death before recovery within 30 days after acute kidney injury onset, stratified by KDIGO stage. Recovery was defined as a serum creatinine concentration <1.5 times baseline and ≤26.5 μmol/L above baseline. Death before recovery was treated as a competing event.

Among the 483 patients with AKI, 268 (55.5%) had KDIGO Stage 1, 110 (22.8%) Stage 2 and 105 (21.7%) Stage 3 AKI. Within 30 days after AKI onset, recovery rates differed substantially by AKI severity. 219 of 268 patients with Stage 1 AKI recovered (81.7%), compared with 71 of 110 (64.5%) with Stage 2 and 53 of 105 (50.5%) with Stage 3 AKI. Mortality before recovery increased progressively with AKI severity, from 10.4% in Stage 1 (28/268) to 20.9% in Stage 2 (23/110) and 27.6% in Stage 3 (29/105). Cumulative incidence curves demonstrated progressively lower recovery rates and higher mortality before recovery with increasing AKI severity (Figure 4).

## Discussion

In this multicentre cohort of more than 1400 adults with SAB, we characterized the occurrence, timing, severity and recovery of AKI in routine clinical practice and evaluated AKI according to commonly used flucloxacillin dosing regimens. Most AKI episodes developed within the first week after the index blood culture, indicating that kidney injury in SAB occurs predominantly early during the course of infection and treatment. Consistent with recent randomized trials, flucloxacillin remained independently associated with a substantially higher risk of AKI than cefazolin. Although flucloxacillin was associated with a higher incidence of AKI, AKI severity and renal recovery were comparable between treatment groups. Across commonly used dosing regimens, we did not observe meaningful differences in AKI occurrence, timing or severity. Importantly, our findings extend recent randomized evidence by providing detailed information on AKI timing, severity, recovery and the association between flucloxacillin dosing and AKI in a large multicentre real-world SAB population.

The higher AKI incidence observed in our cohort compared with recent randomized trials may partly reflect differences in study populations. Whereas only 30.4% of screened patients were enrolled in the SNAP platform, our study included consecutive SAB patients meeting predefined eligibility criteria.^9^ In addition, baseline creatinine definitions differed between studies: recent trials used creatinine values obtained at or shortly after study inclusion, which may underestimate AKI incidence by incorporating early infection-related kidney injury into the baseline creatinine value.^9,10^ Together, these factors may explain the higher observed AKI incidence compared with recent randomized trials.

Interpretation of the association between flucloxacillin use and AKI requires consideration of antibiotic selection in routine clinical practice. Cefazolin-treated patients constituted a smaller proportion of the cohort, reflecting routine first-line prescribing practices for SAB in the Netherlands. This imbalance limits precision for subgroup analyses, particularly late-onset AKI, where event numbers were low. As in all observational studies, confounding by indication cannot be fully excluded despite adjustment for key markers of disease severity. Cefazolin-treated patients differed in baseline characteristics, with greater chronic comorbidity but lower markers of acute illness severity, making the direction of potential bias difficult to determine. Nonetheless, the association between flucloxacillin use and higher AKI risk remained consistent after multivariable adjustment, suggesting that patient characteristics alone are unlikely to fully explain the observed association. Furthermore, higher AKI incidence rates among flucloxacillin-treated patients persisted after accounting for differences in length of hospital stay and were observed across all participating centres, suggesting that the observed association was not driven solely by differences in follow-up duration or by a single hospital.

Interpretation of AKI incidence requires consideration of baseline creatinine selection. Prior studies have shown that baseline creatinine strategies substantially influence AKI detection and staging and that creatinine values obtained during acute illness may underestimate AKI incidence by already reflecting evolving kidney injury.^2,15,16^ Accordingly, we prioritized pre-admission creatinine values to avoid anchoring baseline renal function to measurements potentially already influenced by early infection-related kidney injury. We used a hierarchical approach incorporating two pre-admission time windows, with age- and sex-based estimation when historical values were unavailable. Historical creatinine measurements obtained months before admission may not reflect kidney function immediately preceding infection, particularly after intervening clinical events. Estimated baseline values may not fully capture chronic kidney disease or changes in renal function before admission. Nevertheless, restricting the analysis to patients with a measured baseline creatinine yielded similar results, supporting the robustness of our findings. Patients who already fulfilled AKI criteria before the index blood culture were excluded in our cohort, as these events were considered unrelated to SAB or subsequent antibiotic exposure.

The predominance of early-onset AKI in our cohort highlights the complex and likely multifactorial nature of kidney injury during SAB. According to the ADQI framework, sepsis-associated AKI includes both direct effects of sepsis and kidney injury arising from sepsis therapies.^5^ Consequently, antibiotic-associated nephrotoxicity should not necessarily be viewed as distinct from sepsis-associated kidney injury. Although most AKI episodes occurred within the first week after the index blood culture, with a median onset of 1.3 days, timing alone does not allow differentiation between sepsis-related and treatment-related mechanisms. Regardless of timing, flucloxacillin remained independently associated with AKI after adjustment for baseline kidney function and markers of disease severity, suggesting that antibiotic-related nephrotoxicity may contribute to kidney injury during SAB. The very early onset observed in our cohort is not entirely consistent with the classical delayed presentation of flucloxacillin-associated acute interstitial nephritis, indicating that additional or multifactorial mechanisms may be involved.

A novel aspect of this study is the evaluation of flucloxacillin dosing in relation to AKI in SAB, an area that has not been systematically examined in previous cohorts. Across routinely used dosing regimens, we did not observe meaningful differences in AKI frequency, timing or severity, suggesting that reducing the prescribed dose alone may not mitigate the increased AKI risk associated with flucloxacillin. However, several factors may have limited the ability to detect a dose-dependent association. Dosing decisions were based on clinical practice rather than randomization and may therefore reflect patient characteristics not fully captured in this analysis. In addition, dosing was evaluated using the initial prescribed daily dose, while cumulative flucloxacillin exposure could not be reliably assessed across all participating centres. Given the substantial interindividual variability in flucloxacillin exposure, toxicity may relate more closely to cumulative or actual drug exposure than to prescribed dose alone. Future studies incorporating cumulative exposure, drug concentrations or time-dependent pharmacokinetic measures may therefore provide additional insight into the relationship between flucloxacillin exposure and AKI risk. By integrating AKI timing, severity, recovery and flucloxacillin dosing, our study provides a comprehensive characterization of AKI during SAB and offers important context for the interpretation of recent randomized evidence.

## Data Availability

Please contact the corresponding author to discuss access to the dataset. We welcome opportunities to share data and contribute to collaborative analyses.
